# Real-Time Monitoring Electronic Triage Tag System for Improving Survival Rate in Disaster-Induced Mass Casualty Incidents

**DOI:** 10.3390/healthcare9070877

**Published:** 2021-07-13

**Authors:** Ju Young Park

**Affiliations:** College of Nursing, Konyang University, 158 Gwanjedong-ro, Seo-gu, Daejeon 35365, Korea; jypark@konyang.ac.kr; Tel.: +82-42-600-8563

**Keywords:** emergency, mass casualty incidents, emergency medical services, triage, internet of things

## Abstract

This study was conducted to contribute to active disaster response by developing internet of things (IoT)-based vital sign monitoring e-triage tag system to improve the survival rate at disaster mass casualty incidents fields. The model used in this study for developing the e-triage tag system is the rapid prototyping model (RAD). The process comprised six steps: analysis, design, development, evaluation, implementation, and simulation. As a result of detailed assessment of the system design and development by an expert group, areas with the highest score in the triage sensor evaluation were rated “very good”, with 5 points for continuous vital sign data delivery, portability, and robustness. In addition, ease of use, wearability, and electricity consumption were rated 4.8, 4.7, and 4.6 points, respectively. In the triage application evaluation, the speed and utility scored a perfect 5 points, and the reliability and expressiveness were rated 4.9 points and 4.8 points, respectively. This study will contribute significantly to increasing the survival rate via the development of a conceptual prehospital triage for field applications and e-triage tag system implementation.

## 1. Introduction

Owing to climate change, safety insensitivity, industrialization, and globalization, disasters often cause widespread damage. For instance, coronavirus disease 2019 (COVID-19), which was discovered in 2019 as the first disease of its kind, is an emerging infectious disease that was recorded globally in 2021 to have a fatality rate of approximately 2.17% [1]. The digitally automated pre-hospital triage system such as the internet of things (IoT) is capable of classifying a patient’s severity and guiding them to the appropriate healthcare setting. An effective IoT-based triage system would ultimately help free up time for healthcare professionals to focus on more complex tasks and increase the safety of employees and patients with respect to COVID-19. Kerr et al. [2] implemented a tactile sensing and fuzzy triage system for COVID-19. Further, an automated triage system could improve the quality of service and reduce costs due to the misappropriation of resources [3].

Recent disasters have been caused by various factors, and their sizes and forms have been diverse, large-scale, and multifaceted, which has increased the extent of their damage, and a lack of systematic response may result in a national crisis. In Korea, disaster-related research has been growing rapidly, with an annual average increase rate of 52.8% since 2003 [4]. This indicates that technologies using wireless sensor networks are being used to build infrastructure in the disaster and social safety sectors. As such, there are many critics who say that even though Korea has begun incorporating science and technology into disaster management, domestic R&D is focused on SCI-level papers or only studies technology development instead of practical applications [5]. In other words, as is the case with major advanced countries, Korea has the task of proactively utilizing science and technology as a key means of responding to disasters.

In emergency medical systems for disasters, there is interest in developing medical information technology (IT) to rescue disaster victims rather than in responding in the field. In 2003, a system development study [6] was conducted for the computerization of patient triage. The disadvantage of triage computerization is that although it is useful for determining whether discharge or hospitalization is appropriate, it cannot be used to treat patients with severe conditions continuously because it is only a one-time event. Studies have been conducted comparing the compliance of bypassing transport according to the field triage decision scheme between metropolitan and non-metropolitan emergency medical services [7], the consistency of prehospital- and hospital-stage severity assessments for trauma patients [8], the revised trauma score (RTS) at the prehospital and hospital stages [9], and the prehospital triage system (X-MAS) [10].

The U.S. began developing a system to monitor vital signs in disaster fields in 2004. Researchers developed a CodeBlue system that prevents rescuers from being put at risk by not only monitoring multiple patients simultaneously, but also tracking the locations of site rescuers as well as monitoring safe access passages [11]. A wireless information system for medical response in disasters (WIISARD) first response system for rapid triage and recording real-time medical data electronically has been developed and utilized in mass casualty incidents (MCIs) [12]. In addition, studies have focused on developing various electronic triage technologies such as the advanced health and disaster aid network (AID-N) system, which uses low-power electronic triage sensors to monitor the vital signs of patients while offering location-tracking capabilities [13]. Further, in 2007, researchers developed an e-triage tag system with a lightweight AID-N that enables real-time monitoring with a more specific research scope [14]. In Japan, the e-triage tag system is being upgraded to various specifications by directly measuring the respiration rate using a cannula [15]. The development of these information and communications technology (ICT)-based e-triage tag systems can be seen as a preemptive response strategy in the disaster emergency medical system in each country.

Mobile-web-based e-triage tag systems reduce the communication gap between field rescuers and emergency room nurses when patients move from the prehospital to hospital stage [16]. They also increase survival rates compared to the previous paper triage method [16,17]. In a study on the validation of the e-triage tag system in Iran, the triage category was significantly associated with emergency room length of stay, resource use, in-hospital mortality, and patient bills as outcome measures [18]. HopScore, an e-triage tag system at Johns Hopkins University, filters out low-risk patients, resulting in a 58 min reduction in the overall arrival-to-admission decision time as the field rescuers can concentrate on the more severe patients [19]. In addition, the machine-learning-based e-triage system of vital signs, chief compilation, and active medial history classifies the risk and hospitalization potential of Level 3 patients, who are safe but require large amounts of resources while waiting for treatment, more accurately than the subjective and highly variable Emergency Severity Index [20]. In other words, harmonizing the decision making of e-triage tag systems with the clinical judgments made by rescuers will provide several opportunities to improve efficient triage and severity outcomes.

Four systems were developed for MCI response at the pre-hospital stage with corresponding sensors and devices: eTriage-full [15], AID-N (Advanced Health and Disaster Aid Network) [21], WIISARD (Wireless Information System for Medical Response in Disasters) [22], and iRevive [23]. These studies showed that the triage time, patient omission, and duplicate triage ratios were reduced and that the survival rates were improved by accurately tracking the triage status. Thus, the efficiency of rescuer workload reduction and the prevention of delay in medical services to disaster victims provided the basis for the present research.

Therefore, this study was conducted with the objective of contributing to active disaster response by developing an Internet of things (IoT)-based e-triage tag system for vital sign monitoring to improve the survival rates in MCIs due to disasters.

## 2. Methods

### 2.1. Study Design

This was a methodological study focused on developing an IoT-based real-time e-triage tag system for vital sign monitoring in MCIs.

### 2.2. Development Process

**Figure 1:**
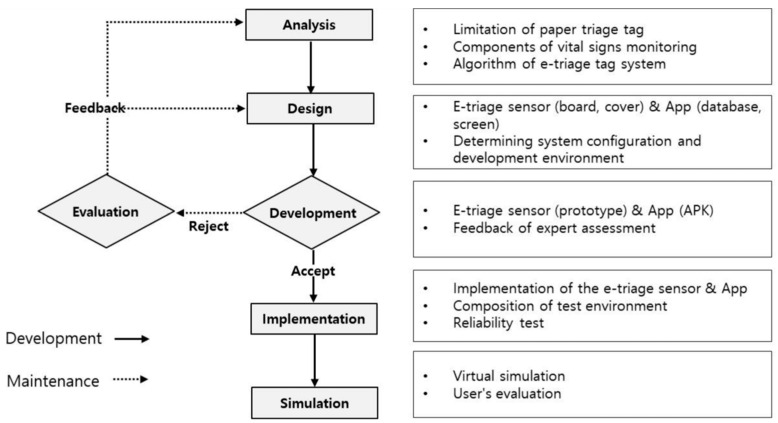
Development Processes of e-triage Tag System.

The model used to develop the e-triage tag system in this study was the rapid prototyping model (RAD) of the software development life cycle (SDLC). The six steps performed in this study were analysis, design, development, evaluation, implementation, and simulation. The development process is shown in Figure 1.

#### 2.2.1. Analysis

At the analysis step, the focus was on developing an algorithm that is scalable by analyzing the literature and the limits of the paper triage tag and subsequently selecting and evaluating the vital sign monitoring elements based on the results obtained from the literature analysis.

#### 2.2.2. Design

In the design step, the development direction and requirements were defined, and the system was designed for the development of e-triage tag system consisting of triage sensor and App.

##### Design of the Triage Sensor

In Japan, eTriage-full measures saturation (SpO2), heart rate (HR), and respiration rate (RR), with Finger Probe and Cannular [15]. AID-N [21] at Johns Hopkins Hospital in the U.S. functioned under very low power constraints and was developed to respond very flexibly to node failures likely to occur at a disaster site using a location tracking system. In addition, the WIISARD [22] in San Diego is an electronic intelligent patient monitoring device that records and documents patient identification and location tracking, patient condition, and medical practice electronically. Its development focused on mobile capabilities that incorporate CalMesh node, Global Positioning System (GPS), mesh and ad hoc networking based on wireless technology to respond to mass casualty incidents caused by terrorist attacks or natural disasters. The key design features include rugged personal digital assistants (PDA) and compact hand-held devices on large screens that are easy to use in field settings. It also features secure data communication over Internet Protocols, simple easy-to-use electronic medical software (EMR) for first responders in a difficult disaster response, battery life of at least 4–6 h, self-diagnosis, and remote software upgrades. Flashing lights of color were designed to flash at the same time as the heartbeat, and locking functions were designed to prevent the patient from operating.

Therefore, in this study, the triage sensor is designed to be easy to wear and simple to measure vital signs.

##### Design of the Triage App

Triage color category is mainly based on the START (Simple triage and rapid treatment) method in medicine. Studies have also constructed color categories for triage app screens based on START [15]. The disaster victim was classified as black (priority 4) if she could not breathe herself after maintaining her airway, green (priority 3) if she could walk, yellow (priority 2) if she could follow a simple command when assessing her consciousness level, or red (priority 1) if the rescuer tried to maintain airway when she could not breathe voluntarily. In a study [13] red (priority 1), yellow (priority 2), green (priority 3), and blue (priority 4) were sorted, and white categories were added if patients were contaminated. It was designed to enable scanning of the patient’s name, address, date of birth, and social security number, but this present study excluded these details in consideration of privacy concerns.

Therefore, the triage app should be able to provide appropriate content for the purpose of the study. Hence, in this study, the app is designed to enable immediate primary screening of patients, such as obtaining their gender [3], level of consciousness, and walkability, and to provide an interface that allows for easy modifications suiting changes in patient status.

#### 2.2.3. Development

Based on the analysis and design steps, the system developer and this researcher developed a prototype triage sensor and the triage app in the form of an APK file. The triage sensor was developed by a group of 10 experts consisting of six emergency medical specialists, a nursing professor with emergency room service experience, a professor at a department of emergency medical service, an emergency room nurse with more than five years’ experience in the emergency room, and a first-degree emergency medical technician working in the emergency room.

The triage sensor board consisted of a sensor block, a charging block, and a Wi-Fi block. The exterior consisted of an initialization button, a green action light-emitting diode (LED), a blue Wi-Fi LED, and a micro universal serial bus (USB) charge terminal. The triage sensor device was developed to display the status in color according to the algorithm category while attached to the finger. For the development of smartphone-linked software, Android apps were developed for calculating SpO2, HR, and RR based on sensing information on the triage sensor device. The full-screen configuration of the triage app used Android Studio 3.5.2, the official integrated development environment (IDE) for Android app development. Using the blocks that make up the designer and program logic of the graphical user interface (GUI) environment, the priority was to automatically match the patient priority on the intro screen. Furthermore, the results of the primary screening items, i.e., gender, level of consciousness, and walkability, were developed in a systematic and accurate manner.

#### 2.2.4. Evaluation

The evaluation step returns to each prior step, i.e., analysis, design, and development, to identify problems and reflect functions by modifying them. The triage sensor was evaluated based on the following criteria [24] (1) ease of use, (2) portability, (3) wearability, (4) robustness, (5) power consumption, (6) provision of continuous bio-signal data. The triage app was evaluated based on a prior study [25] that used the following criteria: (1) speed, (2) reliability, (3) expression, (4) effectiveness. The questionnaire consisted of a total of 10 questions in the Likert scale (1—“not at all”, to 5—“fully so”). The higher the score, the higher the probability of acceptance.

After using the triage sensor and the triage app, the data were collected through a survey. The triage sensor and triage app were modified and supplemented based on the results obtained from the collected data and subjective feedback from experts.

#### 2.2.5. Simulation

In the event of a disaster, the education, research, and projects related to the disaster are clearly important. However, it is difficult to implement this knowledge in the absence of a disaster. Therefore, this study applied and evaluated the e-triage tag systems developed in virtual scenarios. The scenarios were developed based on expert advice, and the number of disaster victims was set at 10, considering the quality of the simulation and the efficient linkage of the triage sensor with the triage app (Table 1).

The scenario was explained for approximately two hours to compensate for the limitations of the simulation and to ensure that the standard patient acting as a disaster victim accurately understood the selected disaster situation. Ten experts who acted as rescuers in the simulation operation described the use of the triage sensor and the triage app, the communication system, personnel and space layout, and control line setting in the event of a disaster. Satisfaction with the reduction of physical burden, psycho-social satisfaction, and reduced patient transfer time were assessed after the simulation. The questionnaire used 1 (very dissatisfied) and 5 (very satisfied) points in the form of a Likert scale: the higher the score, the higher the level of satisfaction with the use of the system. Based on these results, the system was completed after a final modification.

#### 2.2.6. Ethical Considerations

The study protocol was approved by the research ethics committee of the institution before conducting the study. The committee explained to the study participants that participation was voluntary and that they could choose to discontinue participation during the study without any negative repercussions. Written consent to this effect was received.

## 3. Results

### 3.1. Analysis

It was necessary to configure the e-triage tag system to complement the limitations of the existing paper triage tag, enable continuous vital signs monitoring, distinguish the triage color based on an algorithm, and perform data recording and storage. In this study, the following methods were utilized: eTriage-full [15], AID-N [21], WIISARD [22], and iRevive [23].

#### 3.1.1. Limitations of Paper Triage

Paper triage has obvious limitations in terms of continuous patient monitoring and the ability to record essential information such as vital signs and chief complaints manually, especially during treatment. In urgent situations, it can be difficult to read paper triage reports due to handwriting.

The average triage time and death toll with traditional paper triage were previously found to be 28.5 s and 2.23 [15], whereas those with eTriage-full were 15.2 s and 1.4, respectively. According to another study [21], the survival rate was significantly improved to over 90% in the application of AID-N while the control group had a survival rate of less than 10%. In addition, WIISARD accurately tracked the status of triage and reduced the missing triage and duplicate triage ratio [22].

Therefore, field practitioners and researchers must re-examine technical aspects such as e-triage development. In addition, IT-based solutions for patient location, tracking, triage, and information delivery will resolve some of the abovementioned issues and will facilitate overall rescue effort planning. Only when these technical components are added will progress be made.

#### 3.1.2. Evaluation of Vital Sign Monitoring Elements for E-Triage Tag System

For pre-hospital triage, field measurable vital sign factors include temperature, heart rate (HR), blood pressure (BP), respiration rate (RR), saturation of peripheral oxygen (SpO2), capillary refill, consciousness, and electrocardiogram (ECG). SpO2, HR, BP, and EKG are considered to be the most important factors by field rescuers [24]. Therefore, iRevive [23] includes SpO2, BP, and ECG. However, WIISARD [22] consists only of SpO2. As prehospital triage is based on the Simple Triage and Rapid Treatment START method, eTriage-full further incorporates walking, respiration, and consciousness [15]. Meanwhile, for consciousness assessment, the most valuable index is HR [26]. Although the prehospital Korean triage and acuity scale [27] and field triage guidelines [28] recommend the glasgow coma scale (GCS), the study [10] that developed the X-MAS, the proposed triage scale uses alertness, responsiveness to verbal stimulation, responsiveness to pain stimulation, and unresponsiveness (AVPU) to complement technology and time limits.

In these studies, vital sign monitoring was designed for ease of use and carrying, with SpO2, and HR, and additional factors that can be measured without medical equipment prioritized. Therefore, SpO2, HR, and RR were evaluated in this study, which are obtainable through non-invasive measurement in the field, as the most efficient and important factors. These factors were chosen based on a literature review and discussions with relevant researchers and experts. In addition, the basic sensor of the e-triage tag system was designed by adding primary scanning elements to allow field rescuers to evaluate walking, consciousness (AVPU), and gender.

#### 3.1.3. Development of Vital Sign Monitoring Algorithm

AID-N [21] was set to low SpO2 < 90%, bradycardia < HR 60 bpm, tachycardia > HR 100 bpm, and BP (systolic or diastolic change) > ±11% in the vital sign monitoring algorithm. In eTriage-full [15], Category I (red) is HR < 50 bpm or HR > 120 bpm, SpO2 < 90%; Category II (yellow) is HR 50–60 bpm or 100–120 bpm, SpO2 90–96%; and Category III (green) is HR 60–100 bpm, SpO2 96–100%.

In the study [10] in which the prehospital triage scale was developed, the severe level corresponding to immediate care being critical for life included patients with SpO2 < 90%, RR ≤ 30/min, systolic BP < 90 mmHg, severe tachycardia or bradycardia, and a consciousness level of pain response or higher in AVPU. The admission level corresponding to patients requiring hospitalization included patients with SpO2 ≤ 93% in acute cases, systolic BP > 220 mmHg, and a consciousness level greater than or equal to verbal response. Moderate levels that do not require hospitalization but require emergency room care included patients with SpO2 = 92–94%, or with systolic BP > 220 mmHg, but without associated symptoms and with an alert level. Non-emergency levels included patients with RR = 11–19/min, SpO2 > 95%, and normal systolic BP. Meanwhile, while this research [29] does not reveal specific elements for vital sign monitoring, START and sort, assess, lifesaving intervention, and treatment/transport (SALT) were used in the primary triage and the RTS algorithm was applied without modification in the secondary triage.

Therefore, this report suggests that an algorithm should be developed based on the prehospital Korean triage and acuity scale [27] and field triage guidelines [28] and that temperature should be measured separately. The developed algorithm is as shown in Table 2.

Table 3 shows pseudo code of the e-triage rules related to the algorithm outlined in Table 2. To suitably evaluate the e-triage tag system, synthetic data were used to appropriately represent all four outcomes; in the immediate level (red), walking is not possible, SpO2 < 90%, RR < 10/min or > 30/min, and HR < 40 bpm or > 150 bpm. The delayed level (yellow) allows walking, has RR = 20–30 and HR 40–60 bpm or 100–150 bpm. The minor level (green) allows walking and SpO2 > 94%, RR 10–20/min, and HR 60–100 bpm. Decreased level (black) is unresponsive.

### 3.2. Design

The defined development direction requires triage sensors to be easy to wear and simple, and the triage app should be intuitive to use considering reliability and accuracy.

The triage sensor and app are connected to the local network, and each triage sensor is designed to have a fixed IP address assigned and web server running. The triage app calculates SpO2, HR, and RR on the triage sensor through each web server address request.

Message = “Heart rate:” + (String)PulseData(5) + “bpm/SpO2:” + (String)OxiData(5) + “%”;

->Heart rate: 123.45 bpm/SpO2:100%

->Heart rate: _23.45 bpm/SpO2:_98%

->Heart rate: __3.45 bpm/SpO2: __8%

The structure of the information communicated by the triage sensor to the smartphone as follows (it is set to fit the blanks using “_”).

Request: http://192.168.168.(N + 100):80//N = Device_id

Response: “Heart rate” + (String)PulseData(5) + “bpm/SPO2:” + (String)OxiData(5)+”%”;

The functions of the triage app are (a) sensor data analysis: SpO2, HR, and RR; (b) priority analysis; and (c) patient status setting. The data requests and responses are as follows.

SpO2 and HR are calculated from the average of the last 10 datasets, and RR is calculated by dividing the HR average by four (RR = HR/4). Based on this information, the priority is calculated according to algorithms.

The triage app displays the triage sensors of 10 patients on one screen and is designed to move to the next or previous screen by tapping of the screen or pressing of the arrow. The maximum number of screens is 10 (100 patients), owing to router performance limitations. It is designed to return to the initialization state when any of the following is pressed and held: ID, Priority, HR, SpO2, RR, AVPU, Walking, and Gender.

### 3.3. Development

**Figure 2:**
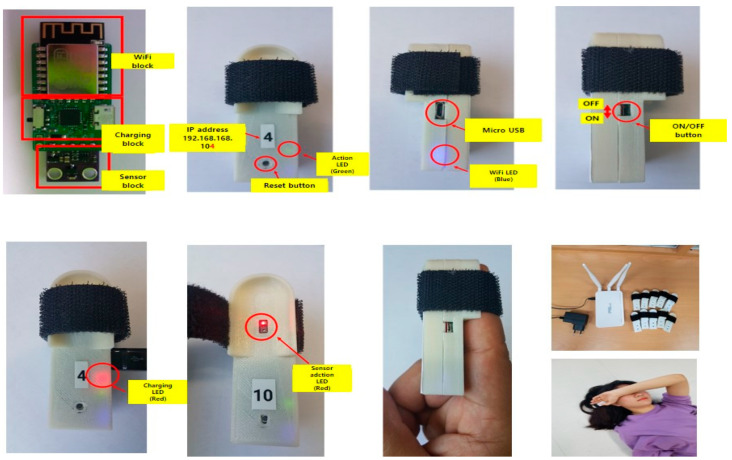
Prototype of Triage Sensor.

**Figure 3:**
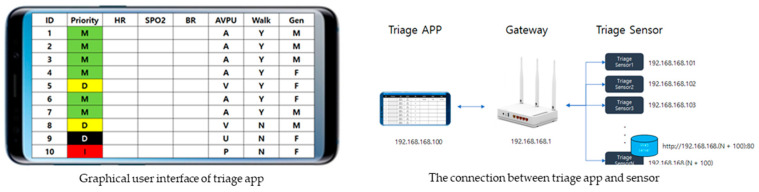
User Graphic Interface of Triage App.

In the early development phase, programming skills necessitated the participation of software engineers and engineering experts. The developed e-triage tag system consists of the triage sensor, triage app, and router. The prototypes of the triage sensor (Figure 2) and app (Figure 3) are as follows.

The triage sensor board was designed using the SolidWorks program 26 (Dassault Systèmes SolidWorks Corp., Waltham, MA, USA) and then printed using a 3D printer [UnionTech (Shanghai Union Technology Co., Ltd., Shanghai, China) RSPro Stereolithography]. It is charged via a micro USB cable, and when it is fully charged, the red LED turns off. As the sensor operates only when it is connected to the router, the LED turns on before it is worn.

### 3.4. Evaluation

After detailed assessment of the system design and development by an expert group, areas with the highest scores in the triage sensor evaluation were rated “very good” with 5 points for continuous vital sign data delivery, portability, and robustness. In addition, ease of use was 4.8 points, wearability 4.7 points, and electricity consumption 4.6 points. In the triage app evaluation, speed and utility scored perfect 5 points, reliability 4.9 points, and expressiveness 4.8 points. Subjective feedback from expert groups indicated that SpO2, HR, and RR were automatically calculated on each triage app linked to the respective triage sensor and shown in the triage color in real time for sensitivity to patient condition changes. However, assuming that the initialization value of the sensor could be incorrect, any information entered within 15 s was ignored and any information entered after 15 s was modified for display. As such, development was confirmed based on expert group assessment.

### 3.5. Implementation

An IP address was assigned to the triage sensor and app, the SSID of the router was set as triage, a secret number was assigned, a wireless connection for each terminal was implemented through the Fing app, and a USB connection was used to debug each sensor. In the reliability test, SpO2 and HR were measured using a Rossmax handheld pulse oximeter (SA210) worn on the left hand and the developed triage sensor on the right hand. The test results showed that each triage sensor had a difference of ±2% or less from the pulse oximeter.

### 3.6. Simulation

After virtual simulations, the effects were identified through surveys of user satisfaction with the real-time IoT-based monitoring e-triage tag system and individual interviews regarding user experience. The virtual scenario was the “Collapse of K University” disaster site that occurred on February 1, 2020. The total number of casualties was 10: 2 red (Priority 1), 6 yellow (Priority 2), 2 green (Priority 3), and 0 black (Priority 4). The simulation results showed that the user satisfaction with the physical burden reduction was 4.3 points, the user satisfaction with psychological and social needs was 5 points, and the time required to checking the patient’s status was 4.8 points.

## 4. Discussion

There should be collaborative response efforts from local firefighters, police, hospitals, and citizens at disaster sites. As the disaster measures provided by the central ministry are practically difficult, it is necessary to develop a field rescue model that can ensure efficient use of medical resources for initial disaster response capabilities. Therefore, this study focused primarily on triage in the field by proposing an e-triage tag system comprising an e-triage sensor and an e-triage app that enables rapid triage of casualties for the establishment of an ICT-based emergency medical system infrastructure. The system was developed through a process of analysis, design, development, evaluation, implementation, and simulation, which will be the focus of this discussion.

In this study, the literature on e-triage tag systems was analyzed to provide the basis for a scalable algorithm by elucidating the limitations of paper triage and selecting and evaluating the vital signs to be measured.

Paper triage involves the use of a color code to determine the severity of patient conditions. Patients classified as red are assumed to require the most immediate attention, followed by yellow, green for those with the least injury, and black for those who have died or are expected to die despite immediate treatment. In the disaster field, re-triage and reassessments are required for rapid transport, but under actual MCI conditions, large numbers of patients cannot be reevaluated, and their conditions may worsen in the meantime. Therefore, the e-triage tag system will help save lives by improving the efficiency of real-time triage and reassessment as well as the overall rescue process.

In the selection of vital sign to monitor, AID-N [21] and iRevive [23] measure SpO2, BP, and ECG while WIISARD [22] measures SpO2. SpO2 is considered useful because it can measure HR and saturation simultaneously. However, rescuers should not triage only through patient vital signs, as disaster victims should be able to make final decisions on priority even in the trial phase. Therefore, elements of walking, respiration, and consciousness (AVPU) were selected, as suggested in eTriage-full [15], and contributed technically to prioritization.

A study surveyed the triage systems in MCI from 1990 to 2018 and showed that 20 different triage systems were used worldwide [30]. It also revealed that to date, no known triage systems and algorithms are good for the clinical outcomes of patients, scene management improvement, or resource allocation. As there is no general worldwide agreement, algorithms must be developed considering specific situations in Korean provincial areas, diverse emergencies and disasters, major patient problems, or resources and facilities needed to respond to patient needs. Therefore, in this study, the easiest and simplest algorithm was selected based on the criteria of the prehospital Korean triage and acuity scale [27] and the guidelines for field triage [28], reflecting the domestic context and opinions of experts who choose to focus on priority.

The analysis phase required the development of an algorithm, which is a key part of e-triage tag system operation. Therefore, the development concept in this study can be classified as a type of preparedness response rather than a type of disaster information provision and emergency contact. In addition to the automatic delivery of priority by an algorithm, treating large numbers of patients in MCIs is an important challenge; hence, few patients can be reevaluated due to the limited number of rescuers. Furthermore, it is based on quantitative data regardless of rescuer bias or stereotypes, in terms of race, gender, ethnicity, or socio-economic status. Thus, the system guarantees fairness.

In the design phase, the triage sensor and app development were focused on the intended development direction and requirements. The initially developed AID-N is GPS-based [21] and has GPS features such as automatic map generation, which is particularly useful in disasters in high-rise or congested urban areas prone to GPS errors [15]. The triage sensor in this study was also designed by incorporating as many IoT functions as possible with GPS-based networking, as overviews of the numbers of patients in different triage areas in disaster fields and clear information about patient locations should be shown to field commanders. Further, medical staff should always be aware of where patients have been transported. The GUI of the triage app requires optimal service delivery in disaster situations; hence, standard software and the best hardware were used to ensure reliability and data accuracy without complexity. Consequently, the design of databases and screens with GPS capabilities ensured efficient information collection.

In the development phase, the triage sensor and app were developed from the outputs of the analysis and design stages. For efficient design as well as the development and evaluation of the system, multidisciplinary participation of disaster field experts and engineering experts was essential. In addition to analyzing the needs through various studies in the development stage, this research was enhanced by an active feedback process involving experts, especially in overcoming software and hardware problems regarding design and development.

In the implementation phase, the e-triage tag system was realized through the triage sensor and app. The time taken for triage before and after implementation of the e-Canadian Triage and Acuity Scale in seven emergency rooms in Ontario, Canada, significantly reduced the numbers of over- or under-triaged patients [31]. Thus, the reliability and accuracy of e-triage tag systems are important indicators. Therefore, it is considered that the obtained triage sensor error of less than ±2% does not limit its usability.

In the evaluation phase, expert assessment of system design and development was performed. The overall satisfaction level of the triage sensor and app was high. The triage sensor was highly satisfactory as it included features that made vital signs easy to measure, was easy to wear, and provided continuous monitoring data. Considering the urgency of disaster situations in the construction of the triage app screen and function, it can be concluded that the user, design, content, and function sides were all satisfied. The system included a function that is briefly configured with information at a glance and changes the priority color in real time via automatic delivery. This is the same context in which a previous study [24] emphasized learnability, familiarity, simplicity, accessibility, customizability, error minimization, and recoverability as user interface design principles for MCI patient-monitoring systems. Therefore, it is anticipated that the e-triage tag system developed in this study will contribute to resolving triage problems arising from the urgency of disaster situations.

In the simulation phase, the ideal system was simulated by predicting various situations and holding in-depth and active meetings. Based on the expert surveys and individual interviews of user experience through virtual simulations, the psycho-social needs scored the highest, and the expectations for shorter transfer times and satisfaction with reduced work burden were relatively high. The results are strongly attributable to the rescuers being able to identify disaster victim vital sign changes directly, and the real-time data storage can sufficiently shortening the triage time in disaster fields.

As the overall goal is to improve disaster survival rates, the health and medical systems sectors should emphasize using proven devices for initial responses in disaster fields. Hence, this research provides a direction for disaster research and calls for systematic responses to disasters. The developed e-triage tag system will be vital as many technical tasks and mechanical devices remain to be integrated into disaster fields.

## 5. Conclusions

This study aimed to significantly increase the disaster survival rate by the development and evaluation of a prehospital e-triage tag system that can be easily applied in the field. Nevertheless, this study is limited to the versatility of mobile devices, such as mobility and GPS location-based characteristics, as very useful tools for responding to disasters. Hence, the potential issues and shortcomings, especially technical limitations, have not been fully considered. For example, smartphone and tablet batteries should last around 12 h, depending on the level of use; however, as more than 24 h of blackouts can occur during disasters, alternatives or backup options should be considered. In addition, large amounts of information will be sent and received in disasters, and responding will take considerable time and manpower; hence, the administrative costs should be considered. If mobile devices are used for disaster victim triage, privacy is a concern as governments or medical institutions can collect and retain personal information and perform data mining on them. This point should also be addressed in future research. Also, all casualties in the study were adults. Future work would include expanding data collection to include a wider range of age ultimately link an in-hospital e-triage system.

As prehospital and hospital-level triage must be of the same standard for effective treatment and reassessment of severity at the hospital stage [32], a study on triage system standardization for e-triage tag system development is proposed.

To reduce the social burden by reducing wastage of medical resources and enhancing effective resource utilization, efficient connection of information and communication between the field and emergency medical institution by sending and storing sensor data on the central server is paramount. In other words, it is proposed to develop a service model that can be commercialized by empirically assessing the development of an infrastructure centered on the concept of tracking.

## Figures and Tables

**Table 1:** The cases of virtual scenarios.

Cases	Diagnosis	Symptoms
Casualties 1	Hip fracture	Lying down, No visible injuries, Quiet, Heart rate 100, Left leg shortened, angled to the left
Casualties 2	Communicated fracture, left tibia	Lying down, Open fracture left lower limb, Quiet, Open fracture left lower limb, Heart rate 130
Casualties 3	Left open fracture, tibia	Lying down, Blood on left leg, Moaning, Heart rate 90, Open fracture left lower limb
Casualties 4	Spine fracture, L1	Lying down, No visible injuries, Crying, Heart rate 80, Severe pain in the lower back
Casualties 5	Left eye penetrating injury	Lying down, Blood in face, Moaning, Heart rate 130, Penetrating injury to left eye
Casualties 6	Open fracture, Lt. lower arm	Walking, Bleeding from left arm, Speak normally, Heart rate 90
Casualties 7	Massive cerebral contusions	Lying down, No visible injuries, Quiet, Heart rate 90
Casualties 8	Hypotension	Lying down, No visible injuries, Quiet, Blood in the abdomen, Heart rate 140
Casualties 9	Wrist fracture	Walking, Blood on left arm, Quiet, Pain right wrist, Heart rate 90
Casualties 10	Psychologic shock	Walking, No visible injuries, Speak normally, Heart rate 70

**Table 2:** Algorithm of e-triage tag system.

Priority	Input	Categories	Range
A & B (Saturation by pulse oximetry, respiratory rate)	SpO2	Dyspnea	<90%
		Moderate dyspnea	90% < SpO2 < 94%
		Normal	>94%
	RR	Bradypnea	<10/m
		Tachypnea	>30/m
		Normal	20/m < RR < 30/m
C (Systolic blood pressure)	SBP	Hypotension	≤90 mmHg
		Hypertension	>220 mmHg
	HR	Bradycardia	<40 bpm
		Moderate bradycardia	40 bpm < HR < 60 bpm
		Tachycardia	>150 bpm
		Moderate tachycardia	100 bpm < HR < 150 bpm
		Normal	60 bpm < HR < 100 bpm
D (AVPU test, walking)	Consciousness	A	Alertness
		V	Responsiveness to verbal stimulation
		P	Responsiveness to Pain stimulation
		U	Unresponsiveness
	Walking	Yes	Possible
		No	Not possible
T (Temperature)	Temperature	Hyperthermia	>38 °C
		Hypothermia	<36 °C
		Normal	36.5 °C < BT < 37.5 °C

SpO2: Saturation by pulse oximetry, RR: Respiratory rate, SBP: Systolic blood pressure, HR: Heart rate, AVPU (A: Alert, V: responsiveness to verbal stimulation, P: responsiveness to pain stimulation, U: unresponsiveness).

**Table 3:** The outputs of the e-triage tag system.

E-Triage Rule	Output	Priority
If (SpO2 is Dyspnea and RR is Bradypnea or Tachypnea) or If (SBP is Hypotension and HR is Bradycardia or Tachycardia) or If (Consciousness is P or U, and Walking is not possible) or If (BT is Hyperthermia or Hypothermia)	Red	Immediate
If (SpO2 is Moderate dyspnea and RR is Normal) or If (SBP is Hypertension and HR is Moderate bradycardia or Moderate tachycardia) or If (Consciousness is A or V, and Walking is not possible) or If (BT is Hyperthermia or Hypothermia)	Yellow	Delayed
If (SpO2 is Normal and RR is Normal) or If (HR is Normal) or If (Consciousness is A and Walking is possible) or If (BT is Normal)	Green	Minor
If (All is unresponsive)	Black	Decreased

## Data Availability

The data presented in this study are available on request from the corresponding author. The data are not publicly available due to privacy or ethical restrictions.
